# Globus Pallidus External Segment Neuron Classification in Freely Moving Rats: A Comparison to Primates

**DOI:** 10.1371/journal.pone.0045421

**Published:** 2012-09-21

**Authors:** Liora Benhamou, Maya Bronfeld, Izhar Bar-Gad, Dana Cohen

**Affiliations:** The Leslie and Susan Gonda Multidisciplinary Brain Research Center, Bar-Ilan University, Ramat-Gan, Israel; Centre national de la recherche scientifique, France

## Abstract

Globus Pallidus external segment (GPe) neurons are well-characterized in behaving primates. Based on their firing properties, these neurons are commonly divided into two distinct groups: high frequency pausers (HFP) and low frequency bursters (LFB). However, no such characterization has been made for behaving rats. The current study characterizes and categorizes extracellularly recorded GPe neurons in freely moving rats, and compares these results to those obtained by extracellular recordings in behaving primates using the same analysis methods. Analysis of our data recorded in rats revealed two distinct neuronal populations exhibiting firing-pattern characteristics that are similar to those obtained in primates. These characteristic firing patterns are conserved between species although the firing rate is significantly lower in rats than in primates. Significant differences in waveform duration and shape were insufficient to create a reliable waveform-based classification in either species. The firing pattern analogy may emphasize conserved processing properties over firing rate per-se. Given the similarity in GPe neuronal activity between human and non-human primates in different pathologies, our results encourage information transfer using complementary studies across species in the GPe to acquire a better understanding of the function of this nucleus in health and disease.

## Introduction

Current thinking emphasizes the role played by the basal ganglia in channeling information from limbic to cognitive and to motor circuits by a parallel and integrative circuit architecture [1]. The central position of the BG network in a neuronal loop connecting most cortical areas primarily to the frontal cortex [2], [3] gives the basal ganglia the potential ability to participate in complex behaviors. However, the role of this structure remains elusive, thus emphasizing the importance of observation of the information flow from the cortex through these nuclei. The primate basal ganglia consist of multiple nuclei: two main input structures – the Striatum (Str) and Subthalamic nucleus (STN) – which are reciprocally connected to the Globus Pallidus external segment (GPe). These three structures converge onto two output structures: the Globus Pallidus internal segment (GPi) and the Substantia Nigra pars reticulata (SNr). Despite differences in terminology (Globus pallidus (GP) and Entopeduncular nucleus (EP) in rats, and GPe and GPi in primates, respectively) and a few structural differences, the rodent and primate basal ganglia roughly share similar cell types and connectivity, suggesting that comparative studies could provide valuable insights. For simplification, we will use the primate terminology also for rodents.

The GPe, located in the core of the basal ganglia, was classically viewed as a relay station along the indirect pathway [4]. Anatomical evidence as well as electrophysiological studies now suggest a more central function for the GPe in the basal ganglia network [5], [6], [7]. The strong reciprocal connections to all basal ganglia input nuclei endow the GPe with capacities to modulate the flow of information through the basal ganglia and its examination should shed light on basal ganglia function in general.

An early electrophysiological study in primates classified GPe neurons into two types based on their distinct *in-vivo* discharge patterns: (1) high-frequency discharge with pauses (85%), and (2) low frequency discharge with bursts (15%) neurons [8]. These two populations are now broadly known as high frequency pausers (HFP) and low frequency bursters (LFB). In addition, a third type dubbed border cells has been described and is believed to represent an extension of the cholinergic neurons of the substantia innominata or nucleus basalis of Meynert [8], [9]. Similar GPe neuronal groups have been reported in humans [10]. Later studies in primates classified GPe neurons based on LFB and HFP firing patterns divergences [11], [12], [13], [14], [15]. In the rodent GPe, the distinction between subpopulations is more controversial. *In-vitro* electrophysiological [16], [17], [18], [19] and anatomical studies [5], [20], [21] have reported conflicting data, with two to three GPe neuron types in each field. A physiological and computational study has suggested that GPe neuron properties are spread over a continuous space, making differentiation into subgroups impossible [22].

Chronic recording is well-developed in rats and permits stable recording of neurons from deep structures for prolonged periods of time. Thus, investigating the GPe in behaving rats could supply further information on the function of the GPe to supplement existing findings in the primate. Creating a common mapping of rat and primate GPe neurons would enable complementary studies within both species. This would allow researchers to benefit from findings on each species while minimizing the limitations inherent to each. Given the interest in observing the information flow through basal ganglia nuclei and the lack of consensus regarding the categorization of rat GPe neurons and their relation to categorization in primates, the current study was designed to provide a classification of GPe cells in rats by drawing parallels between extracellularly recorded GPe neurons in behaving primates and freely moving rats.

## Methods

### Surgucal Procedures and Data Collection

#### Rats

All procedures were in accordance with the National Institutes of Health Guide for the Care and Use of Laboratory Animals and the Bar-Ilan University Guidelines for the Use and Care of Laboratory Animals in Research. All procedures were approved and supervised by the Institutional Animal Care and Use Committee (IACUC). This procedure was approved by the National Committee for Experiments in Laboratory Animals at the Ministry of Health (permit number 01-01-10). Activity of Globus Pallidus neurons was recorded in three freely moving adult male Long-Evans rats alternating between periods of immobility and exploration of the recording cage. The surgical procedure has been described previously [23], [24]. In brief, adult male Long-Evans rats (Harlan) weighing 435 g on average, (range: 415 to 445 g) were sedated with 5% isoflurane and then injected i.m. with ketamine HCl and xylazine HCl (100 and 10 mg/kg, respectively). Supplementary injections of xylazine and ketamine were administered as required. The rat's head was fixed in a stereotaxic frame (Kopf Instruments, USA). After sterilization of the skin, an incision was made in order to expose the skull surface. Connective tissue was removed and the skull surface cleaned. Two craniotomies, slightly larger than the electrode, were made bilaterally above the GPe (AP: −1.4, ML: 3.6, DV: −6.6). 2×8 electrode arrays made with isonel coated tungsten microwires (50 microns diameter – California Fine Wire Company) or 27 gauge cannulae filled with 8 Formvar coated Nichrome wires (coated: 0.0015″, A–M Systems, Inc.) were slowly introduced into the GPe (impedance 0.1–0.2 MΩ at 1 kHz). Electrodes were fixed in place using dental cement, leaving the upper part of the connectors exposed.

At the end of the experiment, the rats were anesthetized with ketamine HCl, xylazine HCl and morphine (100 and 10 mg/kg and 0.15 ml/kg, respectively), and electrolytic lesions were made before perfusion with 10% formalin, brain fixation with 20% sucrose and formalin followed by cryostat sectioning of 60 µm thick slices. Electrode placement was confirmed histologically with a microscope (Nikon Eclipse E400, 1×/0.04).

Following about 10 days of recovery from surgery the animals were connected to the recording system. Neural activity was amplified, band-pass filtered at 150–8000 Hz and sampled at 40 KHz using a multichannel acquisition processor system (MAP system; Plexon Inc, Dallas, TX, USA). All waveforms exceeding a selected threshold were saved and offline sorted for later analysis. Most of the channels containing neurons were also recorded continuously at the same sampling rate to enable additional assurance of single neurons' quality. Offline sorting was performed on all continuously recorded units (OfflineSorter V2.8.8; Plexon, Dallas, TX) and the data were analyzed using custom-written MATLAB software (R2010b, MathWorks Inc., Natick, MA). The animals' activity was continuously monitored in the chamber to ensure that throughout recordings they remain awake.

#### Monkeys

All procedures followed the National Institutes of Health Guide for the Care and Use of Laboratory Animals, Bar-Ilan University Guidelines for the Use and Care of Laboratory Animals in Research and in accordance with the recommendations of the Weatherall Report. All procedures were approved and supervised by the Institutional Animal Care and Use Committee (IACUC). This procedure was approved by the National Committee for Experiments in Laboratory Animals at the Ministry of Health (permit number 18-07-08). Data were obtained from two male cynomolgus monkeys (Macaca fascicularis). The monkeys were kept in an enriched environment under fixed day/night light cycle. During the training and recording periods the animals had free food and were under water restriction. They received their daily water during the experimental session and were supplemented as required following the session. The monkeys' water, food consumption and weight were measured daily and their health was monitored by a veterinarian. Full details of the surgery and recording procedures have been provided previously [25]. Briefly, the monkeys underwent a surgical procedure to attach a recording chamber to the skull allowing access to the GPe and other cortical and basal ganglia structures. The surgical procedure was performed under aseptic conditions and general anesthesia induced by intramuscular ketamine-HCl (10 mg/kg) and Domitor (0.1 mg/kg) and maintained by isoflurane (1–3%), N_2_O (1%) and oxygen (1%) ventilation delivered through tracheal intubation. Appropriate analgesics and antibiotics were given during surgery and postoperatively as required. All surgeries and follow-ups were under the supervision of a veterinarian. All efforts were made to minimize suffering. Recording sessions began after recovery from surgery. The monkeys were seated in a primate chair with their head fixed during the recording sessions. Using a cylindrical guide, eight glass-coated tungsten microelectrodes (impedance 0.2–0.7 MΩ at 1 kHz) were advanced separately into the GP. The electrode signal was continuously sampled at 40 kHz (Alphamap 10.10, Alpha-Omega Engineering), amplified (×1000) and wide bandpass filtered (2–8000 Hz four-pole Butterworth filter) (MCP-Plus 4.10, Alpha-Omega Engineering). Action potentials of individual neurons were sorted offline (OfflineSorter V2.8.7; Plexon, Dallas, TX). The external (GPe) and internal (GPi) segments were distinguished online based on characteristics of neuronal activity and the existence of border cells and white matter between the two segments. Only high-frequency pausers (HFP) and low-frequency bursters (LFB) from the GPe, identified as single neurons using off-line sorter, were included in this study. The average recording time per neuron was 120±47 seconds (mean±STD).

Following the end of the experiment, animals were anesthetized with ketamine (10 mg/kg) and stereotactic marking microlesions (DC current 60 µA for 30 s) were made. The lesions were targeted to dorsal white matter tracts at the anatomical plane that was derived from electrophysiological mapping to be consistent with the anterior commissure (AC0) position. Animals were then deeply anesthetized using sodium pentobarbital (50 mg/kg) and transcardially perfused with 1 liter of physiological saline, followed by 1 liter of 4% paraformaldehyde. The whole brain was removed and buffered in graded sucrose solution 10–30% over 7 days. The brain was then frozen at −25°C and cut in the coronal plane using a cryostat (Leica Mycrosystems). Each section of interest was mounted onto glass slides and Nissl stained. Contours of brain structures were traced using the digitized images and the anteroposterior position of each injection site was plotted on coronal planes, taking AC0 as the origin of the system axes.

### Data Analysis

#### Statistical analysis

All the data are presented as the mean ± SEM. Analysis was structured as 2×2 interactions of animals (primates vs. rats) and cell type (HFPs vs. LFBs) evaluated by a N-way analysis of variance (ANOVAN). Multiple comparisons based on a non-parametric test (Kruskal-Wallis test) provided similar results. Comparison of two groups that did not follow a normal distribution was evaluated by the non-parametric Mann-Whitney U-test.

#### Waveform parameters

Waveform parameters consisted of valley width, peak to valley ratio, peak to valley duration, peak and valley amplitudes and zero-cross. The valley is the minimal amplitude time point and the peak is the maximal amplitude time point coming after the valley. Briefly, valley width describes the duration of the extracellular waveform at its half amplitude, the peak to valley ratio is the absolute value of the peak amplitude divided by the valley amplitude and zero-cross describes the time elapsed between the two time points around the valley in which the amplitude equals zero.

#### Firing parameters

Firing parameters including Coefficient of Variation (CV), Fano Factor (FF), firing rate, mode Inter-Spike Interval (ISI) and peri-modal width were calculated. The term coefficient of variation defines the standard deviation of the ISI distribution divided by its mean. The Fano Factor is the variance of the spike count distribution calculated in non-overlapping time windows, divided by its mean (window duration equals the median ISI of every single neuron). The firing rate is the total number of spikes divided by the total recording time (spike count rate). Mode ISI describes the mode value of the ISI distribution using 1 ms precision bins. In addition, in order to estimate the variability of ISIs values around the mode ISI and thereby measure the ISI distribution width, we calculated the peri-modal width which represents the width of the ISI distribution at an ordinate that equals the mode ISI divided by two.

#### Auto- and Cross-Correlations

Autocorrelations and cross-correlation functions were calculated for latencies of 1000 ms (bin equals 1 ms).

The post-spike suppression (PSP) [26] was defined as the earliest latency at which the rate equaled the average firing rate in the autocorrelation.

An Autocorrelation-Form based Category (AFC) was calculated by applying a low-pass filter on the autocorrelation function and counting the number of peaks. If the low-pass autocorrelation function presented one peak, it was identified as a burster and if two peaks were observed it was identified as a pauser. We validated this parameter by comparing the obtained categorization with a classification used in previous studies on primates.

For the cross-correlation functions, upper and lower confidence levels were calculated as follows: mean and standard deviation of the cross-correlograms at time ±4–5 s were calculated. The probability that the signal crosses a specified limit in 1% of the bins over one second in every bin according to the Bonferroni correction for multiple comparisons was calculated in the following manner: 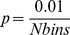. Assuming a normal distribution, we obtained the number of standard deviations (Z-value) required to attain the probability *p* and drew the lower and upper confidence levels at the ordinates corresponding to the mean ± Z standard deviations.

#### Burst analysis

In order to identify bursts in the spike trains, we used the Poisson surprise method [27]. Briefly, the Poisson surprise (*S*) represents the degree to which the occurrence of *n* spikes in time *T* surprises us given the neuron firing rate and assuming a Poisson process. The Poisson surprise is computed in the following manner: *S* = −log*P*, where *P* is the probability that, in a random spike train having the same firing rate (r), a given time interval of length *T* contains *n* or more spikes.
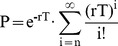



A time interval T was determined for each neuron depending on its firing rate as described below. Initially, an ISI shorter than T was detected. If the following ISI increased the Poisson surprise, it was added to the previously selected ISI until the Poisson surprise did not increase with an additional consecutive ISI. After burst identification, the first spike in the burst was deleted if the Poisson surprise increased by its removal from the burst. Bursts had to contain a minimum of three spikes. The burst percentage counts the number of spikes in bursts compared to the total spikes emitted by the neuron. For example, a neuron containing 20% of its spikes in bursts will obtain a burst percentage of 20%.

The Poisson surprise method requires a criterion for the time interval T to prevent faulty identification of regular ISIs occurring after a long ISI as bursts. The time interval T providing the most reliable identification of bursts by avoiding over or under-inclusion of ISIs [28] was the mean firing rate divided by 2.

#### Pause analysis

In order to identify pauses, the pause surprise method was used [13]. This method calculates how improbable it is that a number *n* of spikes or less appears in a defined period *T* given the average firing rate *r* and assuming a Poisson process. The Poisson surprise is computed in the following manner: *S* = −log*P*, where *P* is the probability that in a random spike train having the same firing rate (r), a given time interval of length *T* contains *n* or fewer spikes. 
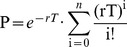
. From all the possible segments formed from the same core interval, the segment that maximizes the pause surprise is called a pause. First, ISIs greater than ten times median ISI were detected (core interval). Then a maximal number of 5 ISIs (upper limit of added intervals) were added one by one before or after each identified long ISI and the pause surprise was calculated. If the addition of an ISI increased the pause surprise, it was included in the pause period. In primates, according to Elias et al. (2007), only periods with a duration greater than 300 ms (minimal length of the final pause) were considered as pauses. Because rat neurons have a significantly lower mean firing rate and the probability of encountering a period of silence of 300 ms is greater than in primates, we increased the minimal duration of the final pause proportionally to the decrease in the mean firing rate compared to primates and set it at 900 ms. Two adjacent pauses were merged if the number of spikes between them did not exceed three (maximal number of spikes enabling merging of adjacent pauses). In rats, the pause fraction represents the number of minutes in which two or more pauses were observed divided by the total recording minutes. Thus, neurons were defined as pausers if they had a minimal pause fraction of 80%. Recordings in primates were shorter (120±47 seconds; average duration ± STD), so a neuron was defined categorically as a pauser if it displayed at least two pauses in the available recording minute without the definition of a pause fraction.

## Results

**Figure 1:**
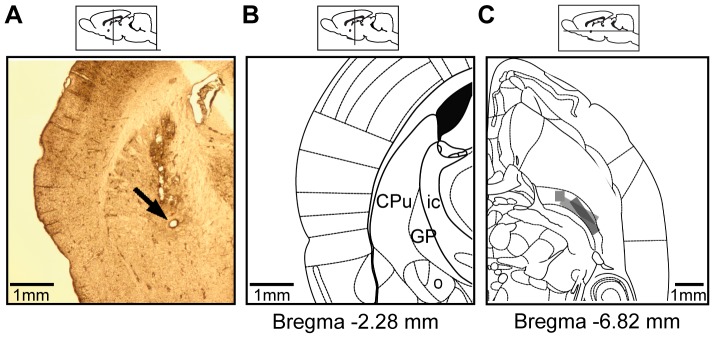
Verification of electrode placement in the Globus Pallidus of rats. A: a 60 micron slice showing electrode placement in a rat GPe following electrolytic lesion. B: Appropriate coronal section from atlas (Bregma: −2.28 mm; [47]). C: Recording sites marked (grey rectangle) for all animals on a planar rat atlas slice (Bregma: −6.82 mm; [47]).

**Figure 2:**
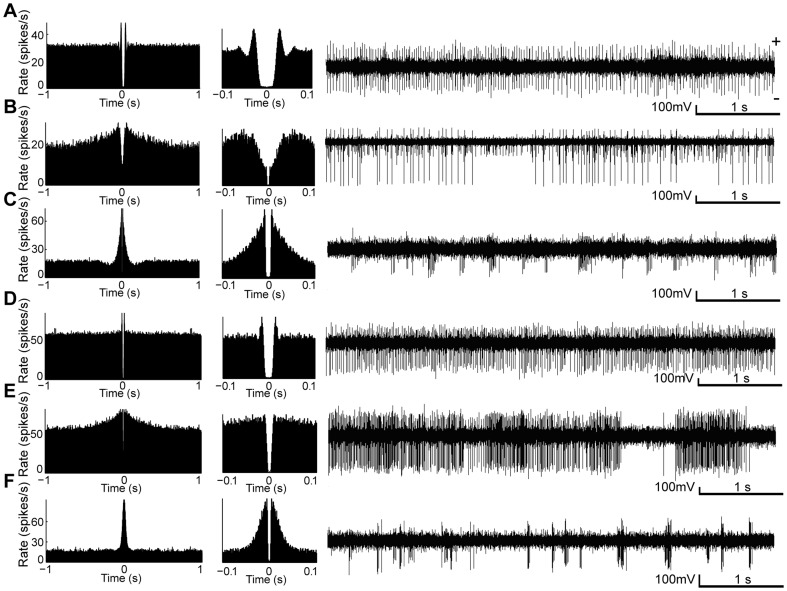
Typical firing patterns observed in rats (A–C) and primates (D–F). Left panel: autocorrelation using a time window of ±1 s, middle panel: autocorrelation with x-axis expanded to ±0.1 s, right panel: spike train of the example neuron. A: example of HFP neuron exhibiting tonic Poisson firing without pauses. B: HFP neuron displaying pauses (pauser). C: LFB neuron (burster). D–F: same type of neurons as in A–C but in primates.

**Figure 3:**
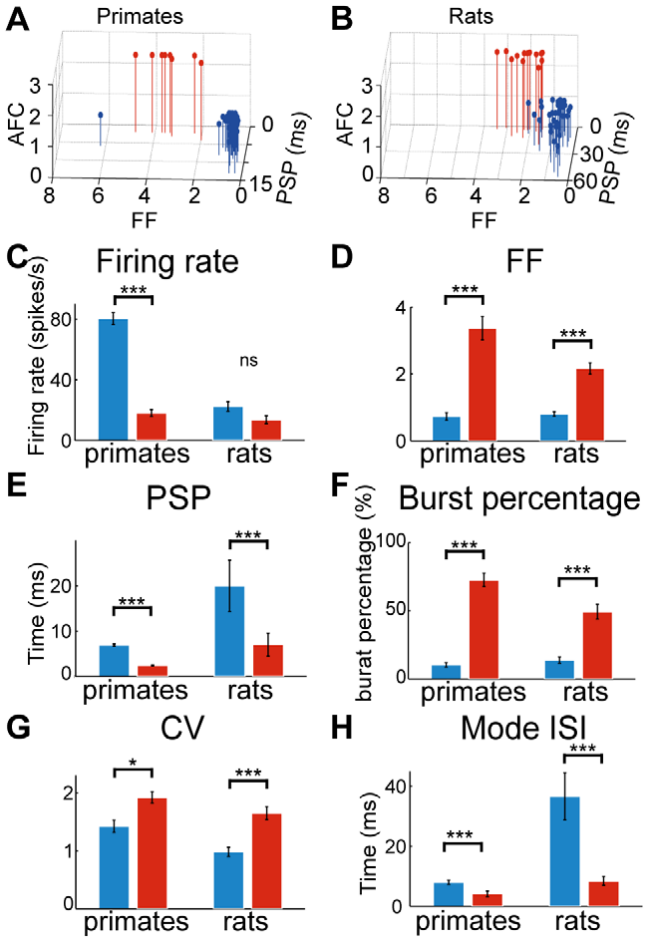
Neuronal classification and firing properties in primates and rats. A and B: 3 dimensional presentation of firing properties leading to the formation of distinct clusters in primates (A) and rats (B). C–H: Bar plots representing parameters of the two groups (HFP - blue, LFB - red) in the two species (left two bars - primates, right two bars - rats).

In order to explore the similarities and differences in rat and primate GPe activity, we characterized and compared the activity of 49 GPe neurons recorded in three rats and 63 GPe neurons recorded in two primates. The position of all electrodes used for recording GPe neurons in rats was verified by electrolytic lesions and histological slice observation (see Methods). An example of electrode positioning in rats is shown in Figure 1A and its corresponding coronal slice is shown in Fig. 1B. A summary of all electrode positioning in rats is shown in Figure 1 C. Initial observation of the recorded spike trains revealed that, on average, firing rates recorded in rats (20.07±2.87 spikes/s) were significantly lower than those recorded in primates (73.37±4.27 spikes/s; Mann Whitney U-test; p<0.001). Nonetheless, two main firing patterns could be distinguished in both species: one consisting of tonic Poisson firing and the other consisting primarily of bursts. Some of the tonically firing neurons also presented periods of silence or pauses. Interestingly, in rats, we did not record neuron with firing patterns resembling border neurons. Autocorrelations and spike trains of rat and primate GPe neurons representative of the three firing patterns types (i.e. low frequency with bursts, higher frequency with and without pauses) are shown in figure 2. We observed a remarkable similarity between rat and primate firing patterns belonging to the same group (compare traces in Fig. 2A & D; 2B & E; and 2C & F). Therefore, we decided to categorize rat GPe neurons based on firing properties, as commonly done in primates. To that end, we measured and calculated a variety of neuronal firing properties such as the coefficient of variation (CV), the Fano factor (FF) and post-spike suppression, and tested whether the two observed populations were also distinct in rats. We found that a data presentation based on firing properties of post-spike suppression, FF and the autocorrelation-form based category (AFC) parameter created two distinct clusters in both rats and primates (Fig. 3A & B). Comparison of the current primate classification into HFPs and LFBs with that obtained earlier on the same data [29] showed a complete match with one exception (∼2%) that exhibited a relatively high value of FF and AFC value of one. Based on the similarity in primate and rat cluster shapes, rat GPe neurons were classified into HFP and LFB. This classification generated 5 outlier neurons that exhibited higher FF values compared to other neurons sharing a similar AFC. By analogy to primate neuronal classification these 5 outliers (5 out of 49; 10%) were also classified as HFP. According to this classification, we obtained 8 (13%) LFB and 55 (87%) HFP neurons in primates and 13 (27%) LFB and 36 (73%) HFP neurons in rats. Based on this classification, similar fraction of pausers was identified within cells classified as HFP neuronal population in rats and primates; 10 out of 36 (28%) HFPs in rats were identified as pausers by the pause analysis and 16 out of 55 (29%) in primates.

Based on the preceding classification, different parameters were calculated and compared between the different GPe neuronal populations (HFP and LFB) found in both species in order to characterize the similarities and differences among these neuronal populations. First, we examined the average firing rates of the identified groups. Primate HFPs exhibited a significantly higher firing rate compared to the LFBs (HFP: 80.28±4.88 spikes/s; LFB: 18.09±1.67 spikes/s; p<0.001; Fig. 3C) whereas in rats the differences in firing rates between HFP and LFB were not significant (HFP: 22.39±3.11 spikes/s; LFB: 13.63±2.56 spikes/s; p = 0.2623; Mann-Whitney U-test; Fig. 3C). By contrast to the firing rates, all parameters characterizing firing patterns properties showed similar statistically significant differences between the two classes of neurons, HFP and LFB, in the two species. These firing pattern differences were maintained between the two species. First, we looked at the parameters used for neuron classification: LFB neurons presented larger FF (rats: 2.17±0.17; primates: 3.37±0.35), a shorter post-spike suppression (rats: 5.47±2.53 ms; primates: 2.43±0.11 ms) and different AFC values than HFP neurons (rats: FF = 0.80±0.07; primates: FF = 0.73±0.11; rats: post-spike suppression = 42.21±5.64 ms; primates: post-spike suppression  = 6.91±0.24 ms) in both species (FF: p<0.001; and post-spike suppression: p<0.001; Fig. 3 D & E). In addition, rat neurons exhibited longer post-spike suppressions than primates (rats: 16.53±1.88 ms; primates: 6.41±2.23 ms; ANOVAN; p<0.001) as can be seen in the representative autocorrelations in Fig. 2 and in Fig. 3E.

Next, we compared additional firing pattern characteristics such as burst fraction, pause fraction, mode ISI and the width of ISI distribution. As expected by their description as bursters, the LFB burst percentage was significantly higher than that of HFP in both species (rats: LFB:49.38±5.37%; HFP:13.90±2.34%; primates: LFB:72.64±4.94%; HFP:10.36±1.61%; main effect, ANOVAN; p<0.001; Fig. 3F). In addition, the mean burst frequency of LFB neurons was 124.9±82.9/min and 238.4±14.9 bursts/min in rats and primates, respectively. LFB neurons were burstier than HFP neurons; consequently their CV was higher than that of the HFPs in both species (rats: LFB: 1.65±0.12; HFP: 0.98±0.08; and primates: LFB: 1.92±0.09; HFP: 1.42±0.10, main effect, cell type, ANOVAN; p<0.001; Fig. 3G). Considering the overlap in firing rate between the HFP and LFB subgroups in rats, we carefully tested whether GPe neurons could make a transition from one mode of operation to the other. None of the recorded neurons displayed a transition between firing pattern typical of HFPs and that typical of LFBs.

As mentioned previously, similar percentages of tonically firing neurons in both rats and primates presented periods of pauses (rats: 28% - 10 out of 36; and primates: 29% - 16 out of 56). The mean pause frequency in the HFP subpopulation of pausers was not significantly different in rats (11.5±4.2 pauses/min) compared to primates (17.0±4.8 pauses/min). Overall, both species exhibited similar fractions of time spent in pauses (rats: 29.9±3.6%, primates: 27.9±2.3%; p>0.9). As expected from bursters, the mode ISI of LFB neurons was significantly shorter than HFP neurons both in rats and in primates (rats: LFB: 8.4±1.5 ms, HFP: 36.7±7.9 ms; p<0.001; primates: LFB: 4.1±1.0 ms, HFP: 8.0±0.7 ms, p<0.01; post-hoc Mann-Whitney U-test; Fig. 3H). In addition, the peri-modal width was significantly smaller in LFB than in HFP neurons (rats: LFB: 15.9±3.5 ms, HFP: 31.4±4.5 ms; p<0.005; primates: LFB: 5.9±1.5 ms, HFP: 10.2±0.5 ms; p<0.05, post-hoc Mann-Whitney U-test). Therefore, we can assume that the mode ISI approximated the intra-burst ISI in LFB neurons, whereas in HFP neurons it represented the most frequent ISI value observed. Conversely, the median ISI was not significantly different between groups (rats: LFB: 52.5±16.1 ms, HFP: 73.3±11.7 ms; primates: LFB: 8.1±1.5 ms, HFP: 10.5±0.9 ms) suggesting that the ISI distribution location itself does not differ between neuronal groups, but they have different forms. Overall, all the firing patterns parameters we looked at exhibited similar group differences among GPe neurons in rats and primates.

**Figure 4:**
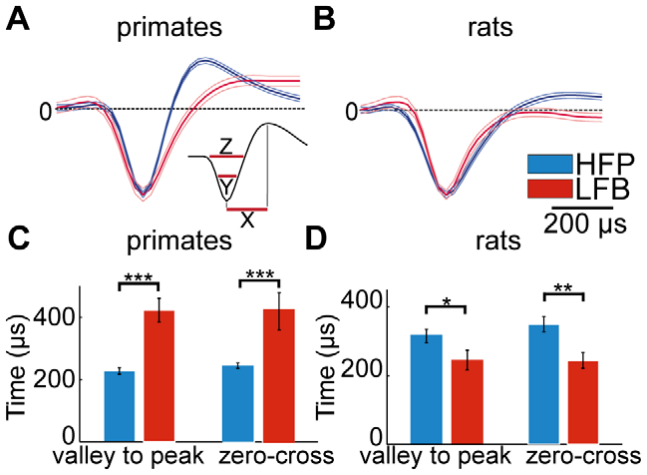
Waveform characteristics. A and B: normalized average waveforms of HFP (blue) and LFB (red) neurons in primates (A) and rats (B). Inset: X represents the valley to peak duration, Y the valley width and Z the zero-cross parameter. C and D Bar plots representing waveform parameters in HFP (blue) and LFB (red) neurons in primates (C) and in rats (D).

We then addressed the similarities and differences observed in various waveform parameters within each species. Unlike neuronal classification in other basal ganglia structures such as the striatum, we were unable to reliably classify the neurons into two distinct populations using rat or primate waveform parameters. However, following the firing pattern based classification, we tested whether the HFPs and the LFBs exhibited distinct waveform parameters in primates (Fig. 4A) and in rats (Fig. 4B). In primates, the mean valley to peak duration of LFB neurons (423.2±37.7 µs) was significantly longer than that of HFP neurons (228.0±10.8 µs; p<0.001, Mann-Whitney U-test; Fig. 4C). The same was observed for the zero cross parameter (LFB: 389.3±54.5 µs; HFP: 220.9±7.9 µs; p<0.001, post-hoc Mann-Whitney U-test; Fig. 4C). In rats, the mean valley to peak duration of LFB neurons (273.1±31.3 µs) was significantly shorter than that of HFP neurons (353.5±21.2 µs; p<0.05, Mann-Whitney U-test; Fig. 4D). This tendency was also observed for the zero cross parameter (LFB: 244.2±22.0 µs; HFP: 350.0±21.5 µs; p<0.01; Mann-Whitney U-test; Fig. 4D).

**Figure 5:**
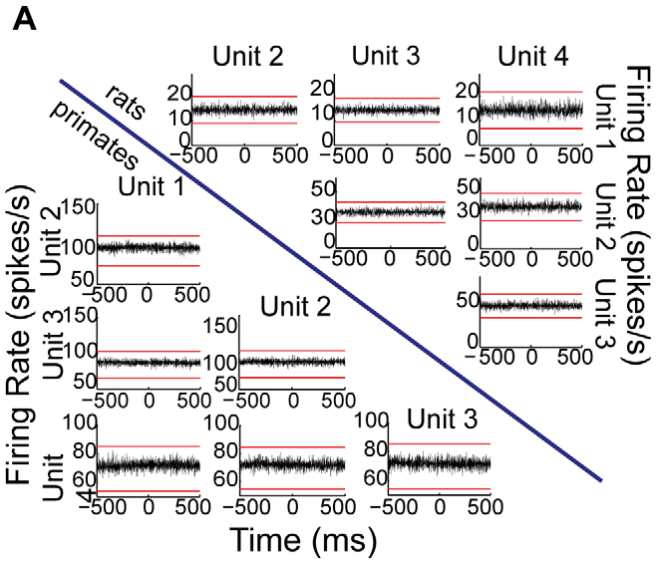
GPe neurons exhibit negligible interactions between pairs of neurons. Cross-correlations in the two species. Below the blue line: cross-correlations during a time window of ±1 s in four units recorded simultaneously in primates. Above the blue line: same in rats. Red lines in every cross-correlogram represent the lower and upper confidence levels (see Methods).

Last, we examined the temporal interactions between neuronal pairs by calculating their cross-correlograms and testing whether different neuronal pairs were significantly correlated. The vast majority (30 out of 31–97%) of rat neuronal pairs exhibited flat cross-correlograms suggesting a lack of interaction between GPe neurons, which is consistent with the primate neuronal pairs which unanimously exhibited flat cross-correlograms (30 out of 30–100%). In rats, the only pair with a significant correlation (LFB and HFP) exhibited a wide peak centered around time 0, pointing to a probable common input that caused correlated changes in firing rate rather than a direct synaptic connection. Overall, neuronal pairs in both rat and primate GPe neurons displayed very little interaction (Fig. 5).

## Discussion

In the present study, we extracellularly recorded GPe neuron activity in rats and primates to compare neuronal activity between the two species and specifically tested whether, as in primates, rat GPe neurons could be categorized as HFPs and LFBs. Our results show that the most striking difference between the two species is the neuronal firing rate, which is extremely high in primate HFPs compared to rats. Most primate studies report high firing rates of about 50 to 80 spikes/s [30], [31] within a wide range of individual cell rates [32]. It appears that HFP neurons in rats exhibit a shifted range of activation and consequently a slower maximal firing rate (range of 2 to 74 spikes/s). This has been reported elsewhere [33], [34], [35], [36] and likely reflects interspecies functional differences rather than major differences in GPe information processing strategies and capabilities. Supporting evidence for this view comes from a similar phenomenon observed in cerebellar Purkinje cells, in which rats and mice [37], [38] show slower simple spike firing rates than primates [39] (approximately 40 spikes/s vs. 80 spikes/s on average, respectively). Waveform parameters in the two species were insufficient to create distinct subpopulations of GPe neurons in both species. Classification of the rat neuronal population based on firing pattern divergence according to the conventional division in primates since the beginning of basal ganglia electrophysiology [8] resulted in the formation of two groups: HFPs and LFBs. These subpopulations represented a proportion of 73.5% and 26.5% respectively in rats, similar to previous intracellular studies [16], [18]. Importantly, previous studies in primates have characterized a proportion of 85% of HFP and 15% LFB neurons [8], [15], [19]. These numbers may be skewed by interspecies differences, the fact that acute experiments entail biased neuronal sampling towards the high-frequency neurons over the more quiescent neurons, or deliberate bias due to lack of scientific interest in the LFB neurons. It remains to be determined whether and how the difference in group fractions influences basal ganglia information processing.

Other diverging parameters observed in this study, such as LFB waveform duration compared to HFP neurons in rats and group fractions are in line with previous electrophysiological studies in rats [18], [20]. Of special interest, previous *in-vitro* studies in rats obtained slight inter-population differences [18], [19]. Our *in-vivo* results reveal that the same parameters (waveform and firing rate) could not be used to categorize GPe neurons into distinct subgroups and may not be conserved in the two species. In contrast, firing patterns led to a similar classification compared to primates; thus firing patterns *in-vivo* differ from those observed *in-vitro* and were the main basis of GPe neuron differentiation into subpopulations. In contrast to the observed firing rates, waveform characteristics and group fractions that differed between rats and primates, all of the firing pattern characteristics measured in the rat HFPs and LFBs showed similar properties typical of primate HFPs and LFBs. These characteristics reflect firing pattern differentiators indicative of different modes of operation employed by the two cell types. We believe that their similarity in rats and primates likely emphasizes conservation of GPe processing properties which are fundamental to normal basal ganglia function. Moreover, measuring the interactions between pairs of GPe neurons in rats revealed negligible correlations between neurons in this structure. Similarly, negligible interactions of less than 5% have previously been reported between primate GPe neurons [40], [41] regardless of inter-neuronal distance [11]. This lack of interaction between GPe neurons supports the idea of functionally independent processing pathways within the GPe that has been preserved over the two species [42], [43], [44]. This fascinating property, along with the anatomical connectivity features of the basal ganglia, give rise to many questions about the processing properties (input/output organization) of the GPe and the basal ganglia in general [45].

Given the similarities and differences found in this study between and within rat and primate GPe neurons, we suggest that as in primates, rat GPe neurons can be reliably divided into two subgroups of cell types: HFPs and LFBs. This conclusion is based on the neuronal firing patterns which capture the differences between the two cell types to a greater extent than other parameters such as waveform characteristics and firing rates which do not support such a categorization. Interestingly, all the parameters affording a clear separation of rat GPe neurons into two cell types are conserved between primates and rats, thus supporting the notion that they are important for normal GPe processing. In contrast, parameters that did not allow for a clear distinction between the two cell types are not conserved between the two species. Specifically, waveform shapes and percentages in the population could arise from technical differences in the recording device and, in any case, are unlikely to directly influence the GPe mode of operation. Nonetheless, the substantial difference in the firing rates between primate and rat HFPs calls for further investigation to determine whether and how the firing rate influences GPe function during behavior.

From an evolutionary perspective, it was claimed that basal ganglia circuitry has been conserved as an action selection mechanism in vertebrates [46] that evolved through reuse of existing ancestral structures. Thus we could expect to encounter similar neuronal groups in vertebrate basal ganglia. Indeed we found similar neuronal firing patterns in the rat GPe neurons as in primates. Given the known similarity between the same neuronal groups in humans and nonhuman primates and the well-known evolutionary conservation of the basal ganglia it seems likely that human, primate and rat studies could play a complementary role in our understanding of the basal ganglia circuitry. Knowledge of the basal ganglia has increased over the past decades; however, its function remains to be elucidated. Parallel recording of basal ganglia nuclei activity in behaving animals will help determine how the firing patterns and interactions observed during rest are altered during behavior and thus could lead to a better understanding of the basal ganglia network function and organization.

## References

[1] Haber SN (2003) The primate basal ganglia: parallel and integrative networks. J Chem Neuroanat 26: 317–330. 14729134 10.1016/j.jchemneu.2003.10.003

[2] Alexander GE, DeLong MR, Strick PL (1986) Parallel organization of functionally segregated circuits linking basal ganglia and cortex. Annu Rev Neurosci 9: 357–381. 3085570 10.1146/annurev.ne.09.030186.002041

[3] Alexander GE, Crutcher MD (1990) Functional architecture of basal ganglia circuits: neural substrates of parallel processing. Trends Neurosci 13: 266–271. 1695401 10.1016/0166-2236(90)90107-l

[4] Albin RL, Young AB, Penney JB (1989) The functional anatomy of basal ganglia disorders. Trends Neurosci 12: 366–375. 2479133 10.1016/0166-2236(89)90074-x

[5] Kita H, Kitai ST (1994) The morphology of globus pallidus projection neurons in the rat: an intracellular staining study. Brain Res 636: 308–319. 8012814 10.1016/0006-8993(94)91030-8

[6] Levesque M, Parent A (2005) The striatofugal fiber system in primates: a reevaluation of its organization based on single-axon tracing studies. Proc Natl Acad Sci U S A 102: 11888–11893. 16087877 10.1073/pnas.0502710102 PMC1187973

[7] Turner RS, Anderson ME (2005) Context-dependent modulation of movement-related discharge in the primate globus pallidus. J Neurosci 25: 2965–2976. 15772356 10.1523/JNEUROSCI.4036-04.2005 PMC6725146

[8] DeLong MR (1971) Activity of pallidal neurons during movement. J Neurophysiol 34: 414–427. 4997823 10.1152/jn.1971.34.3.414

[9] Richardson RT, DeLong MR (1986) Nucleus basalis of Meynert neuronal activity during a delayed response task in monkey. Brain Res 399: 364–368. 3828770 10.1016/0006-8993(86)91529-5

[10] Hutchison WD, Lozano AM, Davis KD, Saint-Cyr JA, Lang AE, et al (1994) Differential neuronal activity in segments of globus pallidus in Parkinson's disease patients. Neuroreport 5: 1533–1537. 7948856 10.1097/00001756-199407000-00031

[11] Bar-Gad I, Heimer G, Ritov Y, Bergman H (2003) Functional correlations between neighboring neurons in the primate globus pallidus are weak or nonexistent. J Neurosci 23: 4012–4016. 12764086 10.1523/JNEUROSCI.23-10-04012.2003 PMC6741070

[12] Kita H, Nambu A, Kaneda K, Tachibana Y, Takada M (2004) Role of ionotropic glutamatergic and GABAergic inputs on the firing activity of neurons in the external pallidum in awake monkeys. J Neurophysiol 92: 3069–3084. 15486427 10.1152/jn.00346.2004

[13] Elias S, Joshua M, Goldberg JA, Heimer G, Arkadir D, et al (2007) Statistical properties of pauses of the high-frequency discharge neurons in the external segment of the globus pallidus. J Neurosci 27: 2525–2538. 17344390 10.1523/JNEUROSCI.4156-06.2007 PMC6672489

[14] Joshua M, Adler A, Rosin B, Vaadia E, Bergman H (2009) Encoding of probabilistic rewarding and aversive events by pallidal and nigral neurons. J Neurophysiol 101: 758–772. 19052110 10.1152/jn.90764.2008

[15] Bronfeld M, Belelovsky K, Bar-Gad I (2011) Spatial and temporal properties of tic-related neuronal activity in the cortico-basal ganglia loop. J Neurosci 31: 8713–8721. 21677155 10.1523/JNEUROSCI.0195-11.2011 PMC6622951

[16] Kita H, Kitai ST (1991) Intracellular study of rat globus pallidus neurons: membrane properties and responses to neostriatal, subthalamic and nigral stimulation. Brain Res 564: 296–305. 1810628 10.1016/0006-8993(91)91466-e

[17] Nambu A, Llinas R (1994) Electrophysiology of globus pallidus neurons in vitro. J Neurophysiol 72: 1127–1139. 7807199 10.1152/jn.1994.72.3.1127

[18] Cooper AJ, Stanford IM (2000) Electrophysiological and morphological characteristics of three subtypes of rat globus pallidus neurone in vitro. J Physiol 527 Pt 2: 291–304. 10970430 10.1111/j.1469-7793.2000.t01-1-00291.x PMC2270075

[19] Bugaysen J, Bronfeld M, Tischler H, Bar-Gad I, Korngreen A (2010) Electrophysiological characteristics of globus pallidus neurons. PLoS One 5: e12001. 20700458 10.1371/journal.pone.0012001 PMC2917366

[20] Millhouse OE (1986) Pallidal neurons in the rat. J Comp Neurol 254: 209–227. 2432103 10.1002/cne.902540206

[21] Nambu A, Llinas R (1997) Morphology of globus pallidus neurons: its correlation with electrophysiology in guinea pig brain slices. J Comp Neurol 377: 85–94. 8986874

[22] Gunay C, Edgerton JR, Jaeger D (2008) Channel density distributions explain spiking variability in the globus pallidus: a combined physiology and computer simulation database approach. J Neurosci 28: 7476–7491. 18650326 10.1523/JNEUROSCI.4198-07.2008 PMC5771640

[23] Nicolelis MA, Ghazanfar AA, Faggin BM, Votaw S, Oliveira LM (1997) Reconstructing the engram: simultaneous, multisite, many single neuron recordings. Neuron 18: 529–537. 9136763 10.1016/s0896-6273(00)80295-0

[24] Jacobson GA, Lev I, Yarom Y, Cohen D (2009) Invariant phase structure of olivo-cerebellar oscillations and its putative role in temporal pattern generation. Proc Natl Acad Sci U S A 106: 3579–3584. 19208809 10.1073/pnas.0806661106 PMC2651350

[25] Erez Y, Czitron H, McCairn K, Belelovsky K, Bar-Gad I (2009) Short-term depression of synaptic transmission during stimulation in the globus pallidus of 1-methyl-4-phenyl-1,2,3,6-tetrahydropyridine-treated primates. J Neurosci 29: 7797–7802. 19535591 10.1523/JNEUROSCI.0401-09.2009 PMC6665635

[26] Schmitzer-Torbert NC, Redish AD (2008) Task-dependent encoding of space and events by striatal neurons is dependent on neural subtype. Neuroscience 153: 349–360. 18406064 10.1016/j.neuroscience.2008.01.081 PMC4206194

[27] Legendy CR, Salcman M (1985) Bursts and recurrences of bursts in the spike trains of spontaneously active striate cortex neurons. J Neurophysiol 53: 926–939. 3998798 10.1152/jn.1985.53.4.926

[28] Cocatre-Zilgien JH, Delcomyn F (1992) Identification of bursts in spike trains. J Neurosci Methods 41: 19–30. 1578899 10.1016/0165-0270(92)90120-3

[29] Bronfeld M, Belelovsky K, Erez Y, Bugaysen J, Korngreen A, et al (2010) Bicuculline-induced chorea manifests in focal rather than globalized abnormalities in the activation of the external and internal globus pallidus. J Neurophysiol 104: 3261–3275. 20592118 10.1152/jn.00093.2010

[30] Hashimoto T, Elder CM, Okun MS, Patrick SK, Vitek JL (2003) Stimulation of the subthalamic nucleus changes the firing pattern of pallidal neurons. J Neurosci 23: 1916–1923. 12629196 10.1523/JNEUROSCI.23-05-01916.2003 PMC6741976

[31] Goldberg JA, Bergman H (2011) Computational physiology of the neural networks of the primate globus pallidus: function and dysfunction. Neuroscience 198: 171–192. 21925240 10.1016/j.neuroscience.2011.08.068

[32] Adler A, Katabi S, Finkes I, Israel Z, Prut Y, et al (2012) Temporal convergence of dynamic cell assemblies in the striato-pallidal network. J Neurosci 32: 2473–2484. 22396421 10.1523/JNEUROSCI.4830-11.2012 PMC6621802

[33] Kita H, Kita T (2011) Role of Striatum in the Pause and Burst Generation in the Globus Pallidus of 6-OHDA-Treated Rats. Front Syst Neurosci 5: 42. 21713126 10.3389/fnsys.2011.00042 PMC3113166

[34] Gardiner TW, Kitai ST (1992) Single-unit activity in the globus pallidus and neostriatum of the rat during performance of a trained head movement. Exp Brain Res 88: 517–530. 1587313 10.1007/BF00228181

[35] Gage GJ, Stoetzner CR, Wiltschko AB, Berke JD (2010) Selective activation of striatal fast-spiking interneurons during choice execution. Neuron 67: 466–479. 20696383 10.1016/j.neuron.2010.06.034 PMC2920892

[36] Chang JY, Shi LH, Luo F, Woodward DJ (2006) Neural responses in multiple basal ganglia regions following unilateral dopamine depletion in behaving rats performing a treadmill locomotion task. Exp Brain Res 172: 193–207. 16369786 10.1007/s00221-005-0312-7

[37] de Solages C, Szapiro G, Brunel N, Hakim V, Isope P, et al (2008) High-frequency organization and synchrony of activity in the purkinje cell layer of the cerebellum. Neuron 58: 775–788. 18549788 10.1016/j.neuron.2008.05.008

[38] Shin SL, Hoebeek FE, Schonewille M, De Zeeuw CI, Aertsen A, et al (2007) Regular patterns in cerebellar Purkinje cell simple spike trains. PLoS One 2: e485. 17534435 10.1371/journal.pone.0000485 PMC1868782

[39] Lisberger SG, Fuchs AF (1978) Role of primate flocculus during rapid behavioral modification of vestibuloocular reflex. I. Purkinje cell activity during visually guided horizontal smooth-pursuit eye movements and passive head rotation. J Neurophysiol 41: 733–763. 96225 10.1152/jn.1978.41.3.733

[40] Nini A, Feingold A, Slovin H, Bergman H (1995) Neurons in the globus pallidus do not show correlated activity in the normal monkey, but phase-locked oscillations appear in the MPTP model of parkinsonism. J Neurophysiol 74: 1800–1805. 8989416 10.1152/jn.1995.74.4.1800

[41] Raz A, Vaadia E, Bergman H (2000) Firing patterns and correlations of spontaneous discharge of pallidal neurons in the normal and the tremulous 1-methyl-4-phenyl-1,2,3,6-tetrahydropyridine vervet model of parkinsonism. J Neurosci 20: 8559–8571. 11069964 10.1523/JNEUROSCI.20-22-08559.2000 PMC6773163

[42] Francois C, Yelnik J, Percheron G, Fenelon G (1994) Topographic distribution of the axonal endings from the sensorimotor and associative striatum in the macaque pallidum and substantia nigra. Exp Brain Res 102: 305–318. 7705508 10.1007/BF00227517

[43] Groenewegen HJ, Galis-de Graaf Y, Smeets WJ (1999) Integration and segregation of limbic cortico-striatal loops at the thalamic level: an experimental tracing study in rats. J Chem Neuroanat 16: 167–185. 10422737 10.1016/s0891-0618(99)00009-5

[44] Percheron G, Filion M (1991) Parallel processing in the basal ganglia: up to a point. Trends Neurosci 14: 55–59. 1708537 10.1016/0166-2236(91)90020-u

[45] Bar-Gad I, Morris G, Bergman H (2003) Information processing, dimensionality reduction and reinforcement learning in the basal ganglia. Prog Neurobiol 71: 439–473. 15013228 10.1016/j.pneurobio.2003.12.001

[46] Redgrave P, Prescott TJ, Gurney K (1999) The basal ganglia: a vertebrate solution to the selection problem? Neuroscience 89: 1009–1023. 10362291 10.1016/s0306-4522(98)00319-4

[47] Paxinos G, Watson C (2007) The Rat Brain in Stereotaxic Coordinates. 6th ed., New York: Elsevier.

